# Supplementary material for: Are carbon emissions trading and green financial instruments synergistic? -Comprehensive quantitative research based on content analysis
Source: PLoS One. 2024 Mar 7;19(3):e0298601. doi: 10.1371/journal.pone.0298601 (PMC10919601; doi:10.1371/journal.pone.0298601)
Supplement: S1 Data — (ZIP) [file pone.0298601.s001.zip › Data/地级市Z_F工作报告.zip!/╣π╢1⁄2/╓╨╔╜╩╨/└┤╫╘╣1⁄2╓┌║┼í╛┬φ┐╦╩2╛▌═°í┐_2016.docx]

十二五”时期及2015年工作回顾

　　“十二五”时期，是我市积极应对艰巨复杂形势，取得重大成就的五年。市政府在省委、省政府和市委的正确领导下，在市人大及其常委会和市政协的监督支持下，认真贯彻党的十八大和十八届三中、四中、五中全会精神及习近平总书记系列重要讲话精神，坚持稳中求进工作总基调，主动适应经济发展新常态，牢牢把握发展主动权，聚精会神谋发展，坚定不移调结构，脚踏实地抓改革，全力以赴惠民生，实现了经济社会持续平稳健康发展，胜利完成2015 年和“十二五”时期各项目标任务，建设“三个适宜”更加美丽中山取得新进展。

　　综合实力迈上新台阶。“十二五”期末，预计全市生产总值突破3000亿元，年均增长10.1%；人均生产总值9.4万元，年均增长9.1%；工业增加值1566.2亿元，五年累计6930亿元，是“十一五”时期的1.7倍，年均增长10.4%；服务业增加值1310亿元，是2010年的1.8倍，年均增长9.5%；现代农业稳健增长，村均集体年收入1634万元，是2010年的1.6倍。固定资产投资1055.4亿元，五年累计突破4500亿元，比“十一五”时期增长近1倍，年均增长17.1%；社会消费品零售总额1080亿元，是2010年的1.7倍，年均增长11.5%；外贸出口280亿美元，是2010年的1.2倍，年均增长4.5%；地方一般公共预算收入287.5亿元，比2010年翻一番。城镇化率提高到88.1%。

　　转型升级取得新成效。翠亨新区成为海峡两岸交流基地、粤澳合作示范区以及首批粤港澳服务贸易自由化示范基地，列为省重大战略发展平台。“十二五”时期，三次产业结构从2.7:58:39.3调整为2.3:54.2:43.5，轻重工业比从59.8:40.2调整为55.5:44.5。对外投资规模是“十一五”时期的9倍，境外工业园实现零的突破。全市境内外上市挂牌企业从12家增至45家，净增上规上限企业391家，发展总部企业49家。实施“新三百”战略，龙头企业与产业集群带动效应进一步增强。首个汽车整车项目落地中山。中心商务区建设成效显著。认定服务业新兴业态企业201家，亿元楼宇13座。中山美居产业园成为省电子商务示范基地。建设高标准基本农田17.7万亩，建成农业标准示范基地60个，500亩以上的现代农业园区33个。设立4个市级产业发展平台，火炬-板芙智能制造示范基地被认定为省智能制造示范基地。单位生产总值能耗和二氧化碳排放、主要污染物排放总量预计均超额完成省下达的“十二五”约束性指标。2015年，装备制造业、先进制造业增加值占规模以上工业增加值比重分别提高2.6、1.6个百分点。一般贸易出口首次超过加工贸易。工业技改投资增长69.5%。研发经费占生产总值比重2.5%，位居全省前列。高新技术企业427家，增长近1倍，增速全省第一。新型研发机构、科技企业孵化器分别增至24和27家。发明专利授权量增长96.4%，成为国家知识产权示范城市，中山（灯饰）知识产权快速维权中心成为全国首个单一行业知识产权快速维权机构。成功创建国家级留学人员创业园，博士后科研工作站增至33家。启动众创金融街和“中山创客·众创空间”建设。规模以上工业企业利润总额、全员劳动生产率分别提高4.5%和9.7%。

　　改革开放取得新突破。中山港口岸扩大对外开放获批。与澳门签署游艇自由行合作协议。成为全国物流标准化试点城市。设立跨境电子商务产业园区，跨境电商三大平台即将建成投入使用。名列“电商百佳城市”第5位。全省十大重点培育进口商品交易中心中山占2个。深入推进商事登记制度改革，实施“三证合一”，在全省率先推出电子执照，“十二五”时期净增市场主体9.75万户，办证效率提高65%。成为全省首批“三单管理”试点城市。农村土地承包经营权确权登记率先颁证，建成农村集体资产监管和交易平台。启动公共信用信息管理系统建设，开通“信用中山网”。组织16家市属国企开展股权招商。长江路改造PPP试点项目落地。完成第六轮行政审批制度改革，审批事项取消调整比例超过50%。在全省率先公布行政权责 “三张清单”。建成市级行政服务中心和网上办事大厅，推行审批职能“三集中一分开”改革，审批时限压缩50%以上。在全省率先公布行政审批中介服务事项目录，建成中介服务超市。简政强镇事权改革有序推进，下放镇区1793项行政审批和执法事项。解决南区、西区“一区两制”问题。实行镇区“大部门”体制。实施均衡性转移支付和定向财力转移支付。实行“督考合一”政府绩效管理模式。创建“法治广东建设示范市”，权力运行进一步规范。圆满完成省对口帮扶计划。

　　基础建设得到新提升。城市总规（2010年-2020年）修编基本完成，实施主体功能区规划，建立“三规合一”协调机制。“十二五”时期，投入230亿元推动交通大发展，是“十一五”时期的1.4倍。深中通道项目获得国家立项，深圳、中山两侧连接线动工。广珠西线、翠亨快线、福源路、沙古公路、105国道跨线桥、阜港公路、中环路、省道S268横栏段等建成通车，中开高速等动工建设，广中江高速、古神公路二期、纵四线等加快推进，全市公路通车里程增加近700公里，达2610 公里，每百平方公里公路密度由2010年的106.5公里提高到145公里。广珠城轨建成通车。启动翠亨新区基础设施建设。中山港客运码头东移加快推进。镇际“断头路”全面打通，建成村级公路545公里，1349公里主干农路实现硬底化。智能公交加快推进，建成快速公交首期示范线，公交出行分担率提升至26%。市级土地储备保有量1.98万亩。完成“三旧”改造1.32万亩，处置闲置土地4.31万亩。完成中心城区核心区主干道雨污分流主体工程，镇区雨污分流工程有序推进。全市“供水一盘棋”加快推进，建成张家边大型泵站等8个重点水利工程。南部组团垃圾综合处理基地加快建设。嘉明三期、中山火电上网发电，南部500千伏加林线全面打通，110千伏白鲤、永益变电站顺利投产。

　　生态环境实现新改善。开展全民绿化行动，成功创建国家生态市。主要污染物总量减排和环境保护责任考核连续四年获“优秀”等级。在全省率先建立生态补偿机制，实现“镇镇均有生态示范村”。空气质量持续改善，PM2.5平均值提前两年达标，位居全国74个重点监测城市前列。“十二五”时期，累计淘汰黄标车及老旧车辆10万辆，推广新能源汽车2000辆。476家重点企业完成清洁生产审核。倡导绿色出行，建成慢行绿道751公里，投放公共自行车8890辆，年均使用600多万人次。加强水环境修复治理，综合治理内河涌1052公里，岐江河“一河两岸”整治取得明显成效。城市生活污水处理率、生活垃圾无害化处理率分别达96%和100%。“十二五”时期，投入绿化资金近50亿元，森林蓄积量327.5万立方米，森林覆盖率由2010年的19.1%提高到22.5%。125个村居成功创建秀美村庄。新建树木园等18个公园，改造紫马岭等23个公园，彩虹绿洲公园、金字山公园等建设稳步推进。

　　民生福祉得到新提高。每年省市的十件民生实事均圆满完成。财政民生支出逐年增长，平均占财政支出比重超过68%。预计城镇和农村常住居民人均可支配收入分别年均增长10.1%、11.6%，城乡居民收入比降至1.53：1。居民消费价格指数年均涨幅为2.4%。五年新增就业27.5 万人，城镇登记失业率在2.3%以下。建成创业孵化基地18个。实施大病医疗保险和困难居民重特大疾病医疗救助制度。蝉联国家卫生城市，建成国家级卫生镇15个。居民人均预期寿命78.8岁。建立低保标准自然增长机制，低保标准城乡统一提高到579元。新增保障房近2万套，改造双低家庭及优抚对象危房2480套。建成居家养老服务中心29个，发放高龄老人津贴7000多万元。五年累计投入310.9亿元发展教育。2015年，高考本科和重点本科录取率双双高居全省第一，顺利通过全国义务教育发展基本均衡市督导评估认定，成为全省第三个推进教育现代化先进市。获评国家历史文化名城，成为国家基层综合性文化服务中心试点市。第四次被授予全国文明城市称号。全民修身行动成效显著。体育惠民工程扎实推进，全民健身示范城市试点获国家通报表扬。建成市镇两级食品药品检测体系，市食品药品检验检测中心投入使用。成为全省首个国家肉类蔬菜流通追溯体系建设试点城市。积分制获全国地方政府创新奖，7.2万名务工人员及子女获得积分入户入学入住公租房资格。

　　社会治理取得新进步。推进十大社会治理工程，荣获“全国社会管理综合治理优秀地市”称号。全民参与社会治理系列行动扎实推进，成为全国社会工作服务示范地区。社区建设“2+8+N”模式入选全国社会治理创新十大最佳案例，建成社区综合服务大厅286个。在全省率先建立社区建设协调委员会和特别委员制度。连续5届荣获 “全国社会治安综合治理优秀地市”。成功创建“无医闹城市”，基本实现“无三非”、文化娱乐服务场所“无三害”。禁毒工作获全国先进。成功打造“中山人才节”等品牌，培养引进各类人才约27万人，其中紧缺适用高层次人才571人。获得地方立法权，建成市、镇、村三级公共法律服务实体和24小时线上法律服务平台。行政执法标准化、信息化管理经验在全省推广。在法检两院的支持下，“法治中山”建设迈上新台阶。信访、调解、应急管理等工作有序开展，矛盾化解取得新成效。双拥优抚工作扎实推进。外事侨务港澳工作获全国先进。台务、民族宗教、统计、审计、国防建设、食品药品监管、疫病防控、安全生产、妇女儿童、老龄、残疾人、红十字、气象、人防、档案、方志等各项工作取得新成绩。

　　各位代表！过去五年，是我市成功应对国际金融危机挑战，综合实力实现新跨越的五年；是我市产业结构深刻调整，开启创新发展新征程的五年；是我市城市品质加速优化，生态环境新提升的五年；是我市改革开放深入推进，激发经济社会发展新活力的五年；是我市社会建设全面深化，文化事业取得新发展的五年。这五年转型发展的探索十分艰辛，经验弥足珍贵，成绩来之不易。这是省委、省政府和市委正确领导的结果，是市人大及其常委会、市政协及各民主党派、无党派人士监督支持的结果，是全市干部群众团结奋斗的结果，是海内外各界关心帮助的结果。在此，我代表市政府，向全市广大工人、农民、知识分子、企业家、干部群众，向各民主党派、工商联、各人民团体、无党派人士和社会各界人士，向驻我市解放军和武警官兵、公安民警，向所有关心支持中山发展的老领导、老同志、港澳台同胞、海外侨胞和国内外友人表示衷心感谢！

　　回顾这五年，我们也清醒地认识到，我市经济社会发展还存在不少问题：受国内外经济形势影响，内外需求总体疲软，经济面临较大下行压力；经济增长依赖要素驱动的局面还没有根本转变，新的增长动力尚在培育；资源环境约束日益趋紧，资源碎片化与低效利用并存，土地综合利用效率不高等问题依然突出；基础设施建设和城市管理相对滞后，产城融合、新型城镇化建设的步伐亟待加快；体制机制不够完善，镇村发展不平衡，发展动能有待释放，营商环境仍需改善；公共服务供给有待增加，交通、环境、医疗、社保、教育等民生工作，与百姓的期盼仍有差距；一些公职人员依法行政意识和能力不强，行政效率和服务水平有待提升。我们将本着对人民高度负责的精神，坚持目标导向与问题导向相统一，切实加快解决。

　“十三五”时期展望

　　“十三五”时期，是全面建成小康社会的决胜阶段，是迈上第二个百年奋斗目标新征程的起步时期。综观国内外大势，新一轮科技革命和产业变革蓄势待发，全国全省发展仍处于大有可为的战略机遇期。我市日益突显的区位优势、加快形成的发展新动力、不断壮大的发展新平台，为“十三五”持续发展奠定了坚实基础。与此同时，世界经济在深度调整中曲折复苏，我国经济发展进入新常态，我们处于转型升级攻坚期、动力转换关键期、收入陷阱跨越期，既要勇于面对困难和挑战，又要善于把握难得的发展机遇，切实增强宏观意识、前瞻意识、创新意识，保持战略定力，变压力为动力，化挑战为机遇，以实干开创未来，以创新推动中山持续健康发展。

　　“十三五”时期，政府工作的指导思想是：全面贯彻党的十八大和十八届三中、四中、五中全会精神，深入贯彻习近平总书记系列重要讲话精神，贯彻落实省委十一届五次、六次全会和市委十三届九次、十次全会部署，按照“四个全面”战略布局和“三个定位、两个率先”任务部署，坚持发展为第一要务，牢固树立创新、协调、绿色、开放、共享的发展理念，紧紧围绕建设 “三个适宜”更加美丽中山总目标，以供给侧结构性改革为主攻方向，以提高发展质量和效益为中心，以创新驱动发展为核心战略，以依法治市为保障，以增进民生福祉为根本，统筹推进经济建设、政治建设、文化建设、社会建设和生态文明建设，建成国家创新型城市，全面完成《珠三角规划纲要》的目标任务，确保率先全面建成小康社会，向率先基本实现社会主义现代化迈出坚实步伐。

　　“十三五”时期，我市要贯彻落实《中共中山市委关于制定国民经济和社会发展第十三个五年规划的建议》，实现如下主要目标。

　　—— 率先全面建成小康社会。确立2017年为我市全面建成小康社会的目标年，全面小康综合指数达97%以上。经济保持中高速增长，产业迈向中高端水平，地区生产总值年均增长8.5%左右，力争比全省提前一年实现生产总值、城乡居民人均收入比2010年翻一番。转方式与调结构取得重大进展，城镇化质量持续提升，全民参与的社会治理体系和能力现代化水平进一步提升，人民生活水平普遍提高，就业、教育、医疗、社保、文化等公共服务体系更加健全，食品药品安全监管更加得力，率先实现基本公共服务均等化，社会更加平安文明和谐。

　　—— 基本建立市场化法治化国际化的制度框架。率先在经济社会发展重要领域和关键环节改革上取得决定性成果，各领域基础性制度体系基本形成。社会诚信体系进一步完善。法治中山建设取得重大进展，形成完备的地方性法规和政府规章体系、高效的法治实施体系、严密的法治监督体系。努力建成法制环境规范、投资贸易便利、监管安全高效的国际化营商环境。

　　—— 基本建立开放型区域创新体系。

　　深入实施创新驱动战略，加快建设国家创新型城市。推动科技创新取得重大突破，初步形成开放型区域创新体系和创新型经济形态，到2020年，R&D 经费支出占GDP比重达2.9%，实现创新创业孵化器、新型研发机构、高新技术企业数量比2015年倍增目标。

　　—— 基本建立具有核心竞争力的现代产业体系。以网络化、数字化、智能化拓展发展新空间，建设智造强市、网络强市，促进现代服务业和先进制造业发展水平不断提高，战略性新兴产业快速发展，优势传统产业加速升级，农业现代化取得明显进展，基本建成现代产业体系。加快重大战略平台发展，到2020年，翠亨新区起步区工业总产值达500亿元，成为推动全市发展的重要增长极。

　　—— 基本形成完善的综合交通体系。

　　坚持以交通引领城市发展，构建 “四纵四横”的高速路网和“三环十射”的快速路网框架，加强对内对外交通对接，推进市内轨道建设，优化港口布局。提前谋划和做好深中通道、港珠澳大桥、深茂铁路开通后的应对准备工作，积极迎接“大桥时代”到来，打造珠江西岸综合交通枢纽。

　　—— 基本形成绿色低碳发展新格局。

　　生产方式和生活方式的绿色、低碳水平不断提升。单位生产总值能耗水耗、单位生产总值二氧化碳以及主要污染物排放的控制水平处于全省前列。主体功能区布局和生态安全屏障逐步巩固，水环境进一步改善，空气质量总体保持良好，森林覆盖率持续提高，争创国家森林城市，建成国家生态文明建设示范市。

　　2016年工作安排

　　2016年是我市“十三五”的开局之年，做好今年工作意义重大。我们将牢记责任和使命，以更宽的眼界、更大的担当，抢抓新机遇，汇聚正能量，励精图治，奋发图强，努力实现“十三五”良好开局，筑牢有利于中山长远发展的基石。

　　2016年主要预期目标是：生产总值增长8%-8.5% ，固定资产投资增长15% 以上，社会消费品零售总额增长10% 以上，出口总值增长1% 以上，一般公共预算收入增长9% 左右，城乡居民人均收入增长与经济增长基本同步，居民消费价格涨幅预期2%左右，城镇登记失业率控制在3.5% 以内，节能减排约束性指标完成省下达年度计划。

　　2016年，我们将以供给侧结构性改革为主攻方向，加快转型升级，做大做强实体经济，提升“三个适宜”城市发展水平，致力社会善治和兜底社会民生，努力让经济社会发展更加优质高效，让创新驱动更加强劲有力，让双向开放更加多元包容，让城市发展更加集约协调，让生态环境更加宜居舒适，让百姓生活更加幸福安康。

　　为实现上述目标，必须做好以下六方面工作。

　　一、坚持提质增效，促进经济稳增快转

　　以提升经济发展的质量和效益为中心，加快盘活存量，做优增量，加强供给侧结构性改革，把去产能、去库存、去杠杆、降成本、补短板落到实处，提供更优质的产品，促进消费模式升级，助推制造业智能化、服务业高端化，保持经济运行在合理区间。

　　扩大有效投资。把握投资的方向和重点，提高投资有效性和精准性。安排市重点项目210项，年度计划投资381亿元。围绕发展“高精尖”装备的产业定位，引进一批高端装备制造项目，重点发展智能制造、光电装备、新能源装备及医疗器械等，保持工作母机企业数量及质量处于全省前列。加快发展新能源、新材料、健康医药等战略性新兴产业。支持企业利用移动互联网、云计算、物联网等新一代网络技术，发展创意经济、文化经济。优化投资服务，推动亿元以上项目落地。实施精准招商，推动一批优质项目落户。深化政府与社会资本合作，用好开发性金融支持政策。加快宽带网络等信息基础设施建设，推进农村电网、智能电网、充电设施建设。发展热电联产、分布式能源。推进11个重点水利工程建设。

　　拓展内外市场。深耕发达国家市场，拓展新兴市场，巩固出口市场份额。推动境内外互设贸易合作平台，在南非、印尼、尼日利亚等国家建设中山产品展销中心。多渠道扩大消费，引导商贸聚集区差异化发展，鼓励连锁商业、便利店、电商企业进社区，推动线上线下融合发展。做好 “一节一促消”主题活动。打造“中山好生活”掌上惠民服务平台。推动展览业市场化，培育一批特色鲜明的品牌展会。支持制造业企业利用电子商务转型，着力发展农村电子商务，推动沙溪、小榄、南头、西区等镇区电子商务发展。促进信息消费，发展网络经济，丰富教育、文化、体育、医疗等民营服务，壮大健康养老消费。

　　推动实体经济发展。建设工业强优城市，探索都市型工业新路子。安排产业扶持资金15亿元。开展降本增效专项行动，千方百计降低企业负担。落实中央和省减税降费政策，进一步减免涉企规费，降低企业生产成本。鼓励发展社会化、集约化、网络化生产性物流，降低企业交易成本。用好15亿元企业融资专项资金，打出政策性担保、助保贷等“组合拳”，降低企业融资成本。加快新型专业镇发展，选择2-3个专业镇，实施“一镇一策”行动计划，打好传统优势产业转型升级攻坚战。大力发展民营经济，破除准入限制、项目审批等制度性障碍。抓好企业上规上限，重点解决骨干企业用地和用人问题。关爱中小微企业健康发展，为困难企业提供分类指导帮扶。加快产业平台开发建设。继续推动工业园区整合提升。建设全国质量强市示范城市。

　　提高要素资源配置效率。优化镇区资源配置，加快高产出区域生产要素投放。加大对欠发达镇区转移支付力度，安排8亿元用于镇区均衡性转移支付，安排12亿元用于镇区基础设施建设补助，安排3亿元用于镇区临时财力补充。实施差别化供给政策，推动土地、环境等宝贵要素与先进生产力高效匹配。整合财政专项资金，盘活存量资产，优化开发融资方式，提高支出效益。运用娱乐体验、时尚文化等要素，改造提升存量商业物业。加大闲置土地处置力度，引导建设单位整合利用碎片用地。深入推进“三旧”改造。加大建设用地收储力度，为城市建设发展提供强力支撑。统筹解决一批土地历史遗留问题。探索建立工业用地“先租后让、弹性年限”出让制度。有序推进锌铁棚改造，盘活闲置工业厂房。科学调控、综合施策，保持房地产平稳健康发展。扩大务工人员居住需求，吸引广深港澳房产投资。发展住房租赁市场，建立购租并举的住房制度。鼓励发展文化地产、旅游地产等新型业态。加强经济运行分析监测，做好政策储备。

　　加快发展现代服务业。以深中通道、港珠澳大桥、深茂铁路规划建设为带动，加强与深港澳对接合作。培育发展服务业新兴业态。大力发展工业设计、商务服务、信息服务等生产性服务业，着力发展家庭服务、健康服务、体育赛事等生活性服务业。推进物流标准化试点，引进培育物流龙头企业，提升物流仓储智能化水平。发展总部经济和楼宇经济，培育壮大一批亿元楼宇。推进服务业集聚区建设。年内规上限上服务业企业净增超百家。

　　推进农业现代化。加快两岸四地现代农业合作示范区建设，打造现代农业发展新平台。市本级财政安排11亿元，用于农业发展。发展特色农业，推动一二三产业融合发展，培育一批上规模、带动性强的现代家庭农场。加快农村综合改革，推进农村土地承包经营权确权登记颁证，探索农民住房、承包经营权抵押，加大金融对农村支持力度。大力发展高效设施农业，推广运用农业物流网等智能技术，创立花木研发中心。建设农业公园和农业综合体。推动连锁经营进社区，培育发展农产品电商。加强农产品质量安全监管，强化重大动植物疫病防控，建成动物检验检测体系。

　　二、坚持创新引领，提升产业核心竞争力

　　实施创新驱动发展核心战略，努力把经济增长动力从要素驱动切换到创新驱动上来，形成一系列创新驱动的制度安排。加大吸引人才力度，获取更多人才红利，发展分享经济，营造有利于大众创业、万众创新的良好环境，让城市充满创新创业活力。

　　加强企业创新能力建设。加强企业研发机构建设，实施企业研发费补助、创新券等政策，高新技术企业净增超100家。推广新型孵化模式，重点建设若干个市级以上新型研发机构，实现孵化器专业镇全覆盖。重点围绕健康医药、先进装备制造等领域实施重大科技专项，支持企业开展重大产业关键共性技术、装备和标准的研发攻关。加快知识产权服务业发展，实现发明专利申请量比2014年翻一番。

　　加大技术改造力度。推动信息化、智能化与工业化深度融合发展。深入实施“互联网+”和大数据行动计划，推进电子政务云、工业云等云服务平台建设。率先在优势传统产业建立智能示范工厂，提升工业机器人、高端数控产品、自动化生产线等智能装备的研发、普及应用水平。设立技术改造设备融资租赁基金。推进火炬-板芙省级智能制造示范基地、小榄智能制造示范镇建设。

　　加快重大创新载体建设。积极参与珠三角国家自主创新示范区建设，建立并完善深中协同创新体系。探索“一园多区”发展模式，强化火炬开发区和翠亨新区的集聚和辐射作用。启动大型科学仪器资源共享平台建设。组建广东省科学院中山分院、国家超级计算广州中心中山分中心，办好工业技术研究院和产业技术研究院。巩固提升市食品药品检验检测中心、南头家电创新中心、小榄和古镇生产力促进中心等公共服务平台，建设一批专业镇协同创新中心和产业技术创新联盟。

　　促进科技金融融合发展。发展科技银行、知识产权质押融资、科技保险、互联网金融等新型金融业态。做大科技信贷风险准备金池，大力发展创新创业投资基金。建设众创金融街和省金融科技产业融合创新试验区。组建市科技金融服务中心，成立政策性融资担保公司，开展互联网股权众筹融资试点。支持企业上市挂牌，鼓励企业发行公司债和私募债。

　　加大产业组织创新力度。推动产业融合，垂直整合中山美居等传统优势产业。鼓励抱团发展，凝聚政府和社会组织帮扶合力，加强区域品牌、行业品牌和联盟标准建设。支持企业并购重组，整合低效产能，培育一批骨干企业。实施“四个一批”战略工程，加快市属国资商业类资产整体上市，推动镇属企业改革，整合资源，提高企业竞争力。建设新型公共服务平台，推动众包研发设计和网络化制造模式。

　　吸引创新人才聚集扎根。全面梳理人才政策，放宽入户条件，简化落户程序，清除一切不利于人才入户的制度障碍。实施海外高层次创新创业人才引进计划、新企业家计划和高技能人才培养计划。加快建设人才创新创业生态园，打造创新企业联盟与人才服务联盟平台。鼓励多方共建新型专业镇“产业（人才）学院”，培养产业集群紧缺适用专业人才。探索开展经营性领域技术入股改革，激发科技人才创新活力。

　　三、坚持深化改革，提升对外开放的质量和水平

　　深化重点领域和关键环节改革，着力形成促进经济社会优化发展的体制机制。实施更加积极主动的对外开放策略，推动对内对外开放相互促进、“引进来”与“走出去”紧密结合，构建更高水平的开放新格局。

　　建设国际化法治化营商环境。深化行政审批改革，完善权责清单制度。推进简政放权，加强事中事后监管。完善市行政服务中心，巩固“三集中一分开”成效。落实“三张清单”。加强“中介超市”管理，规范中介服务市场。拓展网上办事大厅功能，提高网上办理率和办结率。对接国际经济管理先进理念和通行规则，促进办事准则与国际接轨。培育壮大各种中介和专业服务机构。完善社会信用体系，健全失信治理机制。加强市场监管体系建设，加大灯饰等产业市场秩序整治力度。优化企业服务，打通惠企政策“最后一公里”。

　　加快外贸转型升级。推动外贸向优质优价、优进优出转变，引导加工贸易企业加强研发设计、培育自主品牌。推进古镇、南头等国家和省外贸转型升级示范基地建设，建设出口工业品质量安全示范区。完善跨境电商政策扶持体系，打造跨境出口电商产业功能区。发展旅游购物、综合外贸服务等新型外贸模式。加强口岸信息化建设，推行国际贸易“单一窗口”，深化“三互”通关合作，提高通关效率。加快推进中山港口岸扩大开放验收。推广出口退税网上申报和限时办结等制度。

　　推动更高水平 “引进来”和更大步伐“走出去”。支持“走出去”与“引进来”双向投资发展，打造境内外“双平台”互动服务模式，支持企业设立境外工业园区，建设“走出去”服务中心和综合服务信息平台。制定参与国家“一带一路”建设行动计划。争取用好广东丝路基金。大力支持企业开展海外并购，抱团开展国际产能合作，培育一批有影响的跨国企业。发展中瑞（欧）工业园等重大产业平台。发挥侨乡优势，涵养和运用外事侨务港澳资源，推动经贸、文化、旅游、体育等领域对外交流。加强中山驻欧洲等经贸代表处、人才工作站、美国硅谷中山创新创业孵化基地建设。

　　全面加强区域合作。积极对接南沙、前海、横琴自贸区，以最快的速度运用自贸区可复制的政策，推进落实粤港粤澳合作框架协议，加快实现澳门-中山游艇自由行。推进粤港澳服务贸易自由化，推动省级服务外包示范城市建设。开展深中协同发展战略合作，加强产业对接，共建“半小时经济生活圈”。深化与周边城市合作，打通一批市际“断头路”。

　　四、坚持统筹协调，构筑现代城市新格局

　　尊重城市发展规律，加强“五个统筹”，主动融入粤港澳大湾区，优化城市功能布局，转变城市发展方式，完善城市治理体系，形成城市发展新动力，加快建设“精明增长、紧凑集约、高效绿色”型城市。

　　提升城市功能品质。推进新型城镇化“2511”试点建设。启动新一轮城市总体规划（2010年-2030年）修编，推进“三规合一”，形成新的土地管理和项目落地机制。完善主城区、副中心、各大组团的基础设施和公共服务体系，提升“双核两副”的集聚和辐射效应。推动翠亨新区建设上升为国家战略。实施低影响开发建设模式，建设海绵型城市。有序推进地下空间开发试点。加快城市综合管廊建设。推进地籍调查。做好永久基本农田划定。年内80%以上村居建成秀美村庄。加快地质灾害整治。

　　加快交通基础设施建设。动工建设深中通道主体工程，加快中开高速及小榄支线、广中江高速等建设。争取深茂铁路深圳-中山-江门段尽快启动。启动一环、二环贯通工程，动工建设一环长江路、二环东段，推进二环南段（南外环延长线）和北段（沙港公路）、广珠中线二期等。实现中山港大桥扩建、古神公路二期北段等建成通车，改造番中公路、105国道南段、康华路等，动工建设大沙南路、富康北路等。启动市汽车总站升级改造。推进中山港客运码头东移。加快推进城市轨道环评、用地、规划等前期工作，争取建设规划获得国家批复。做好广珠城际站场周边TOD 项目开发。全面完成群众出行“最后一公里”畅通工程。推进公共停车场建设，着力解决医疗机构停车难问题。完善城市慢行系统。

　　创新城市治理方式。加快推进市镇村三级政务服务体系建设，推动社区综合服务大厅规范化和标准化，打造基层公共服务综合平台。完善全民参与社会治理机制，发挥枢纽型组织的引领服务作用，推进中国社会创新（中山）基地、镇区全民公益园等实体平台建设。完善城市管理数字化平台，发展民生服务智慧运用。完善城市微管理制度，建立快速反应机制。优化交通组织方式，加大堵车点治理，建设安全顺畅的通行环境。探索出租车行业改革，规范电动车管理。加强交通道路工程建设和维护的现场管理。统筹镇区相邻地带的开发建设。推进农村社区建设试点。

　　建设休闲旅游城市。加强全市旅游资源的统筹规划和整体设计，构建全域旅游格局。实施“旅游+”工程，推动旅游业与休闲农业、特色工业、健康养生、文化创意、传媒娱乐等业态融合发展，加快形成旅游新业态。力争孙中山故里旅游区建成国家5A级景区，启动红木文化景区、灯饰文化景区创A 计划。建设金钟湖风景区。加强旅游宣传推广，擦亮菊花会、灯光节、特色展会、“周末去哪儿”等旅游品牌。倡导休闲度假游，开发特色旅游商品，完善旅游标识系统，扩大中山旅游的知名度和影响力。

　　五、坚持生态优先，建设碧水蓝天美好家园

　　让绿色低碳成为发展底色，增强生态产品供给能力，构建人与自然和谐相处的良好环境，打造鸟语花香、山清水秀、环境优美的宜居城市。

　　加强环境保护和生态修复。严格落实生态控制线，建立生态环境保护和损害问责制度体系。实施差别化环保准入，坚决淘汰落后产能。严把建设项目审批关，严控“三高”项目上马。加快建筑、交通等领域的节能改造。推进重污染工业园区环保设施改造。加强重点企事业单位碳排放核查，开展镇区碳强度年度目标责任评价考核。加强机动车减排，坚决淘汰“黄标车”，大力推广新能源汽车。加大环保执法监察力度，设立“环保警察”，严厉打击环境违法行为。开展土壤分类管理和污染修复试点示范，实施大气污染防治行动计划。

　　加强水环境综合治理。坚持“截污、清淤、补水”多措并举，大力开展内河涌整治，改善和修复水环境。落实“南粤水更清行动计划”，推进以岐江河为主的水环境综合治理，狠抓跨镇区河流污染整治。实施最严格水资源管理制度，落实水资源用水总量和效率双控行动。严格保护饮用水源，推进南部三镇取水口上移工程。完成主城区雨污分流工程收尾工作，有序推进镇区雨污分流工程。推进水浸黑点整治。

　　创建更多绿色空间。维护城市发展天际线，强化城市空间设计，让市民看得到蓝天白云。启动国家森林城市创建。推进大尖山、蒂峰山、铁炉山等森林公园建设，推动翠亨国家湿地公园、西区彩虹绿洲公园、联石湾湿地公园等一批湿地公园建设，加快古香林公园、金字山公园、树木园三期建设，推进公园儿童体验配套设施建设，加快规划儿童主题公园。扩大城乡绿化，推进景观林、公共绿地和沿河沿路绿化带建设。增加城市公园绿地面积，优化生态空间景观结构。加强绿道网建设，推进城区主干道可视范围废旧石场复绿。

　　六、坚持共建共享，提升人民群众获得感

　　按照“普惠共享、补齐短板”的原则，厘清政府与社会服务边界，引导和鼓励社会资本加大公共服务供给，让人民群众有更大的获得感，加快朝全面小康社会迈进。

　　完善就业社保体系。安排8.3亿元用于医疗、住房等方面社会保障支出。完善医保支付和大病医险制度，保持养老金正常增长，推进省内异地就医即时结算。实施“十百千万”创业工程，加大重点群体就业创业帮扶。健全职工工资稳定增长机制，落实强农惠农富农政策，促进城乡居民收入持续增加。完善医疗救助和临时困难救助制度，提高低保标准和补助水平。实施双低家庭及优抚对象危房改造工程。健全住房保障政策体系。制定高龄、失能老人补贴制度，全面完善“居家养老为基础、社区养老为依托、机构养老为支撑”的养老服务体系。

　　提供更加公平优质的教育。实施学前教育普惠工程，提升公益普惠性幼儿园办学水平。推进义务教育均衡发展。推动普高集约化发展。加快中职教育全市统筹发展，推进职业教育综合改革。促进民办教育规范、特色发展。以教育信息化全面推动教育现代化。大力发展高等教育，争取国内外知名理工科大学落地中山。加强师资队伍建设，提高教师教书育人水平。发展家庭教育。放宽积分入学条件。

　　发展医疗卫生事业。启动公立医院综合改革，建立维护公益性、调动积极性、保障可持续性的运行新机制。深化“医联体”试点，加快形成基层首诊、双向转诊、急慢分治、上下联动的就医秩序。采取综合政策，放宽准入，加快社会办医发展。实施医师多点执业和区域注册制。建立健全住院医师规范化培训制度，推进家庭医生签约式服务。实施“全面二孩”政策，扎实推进妇幼健康服务。加强传染病、慢性病、地方病等重大疾病综合防控和职业病危害防治。提升中医医疗、中西融合与“治未病”服务能力。加强医德医风建设，实施改善医疗服务行动。

　　扎实推进文化体育建设。办好孙中山诞辰150周年系列纪念活动。推进中山纪念图书馆、市博物馆群、小榄文体中心等文化项目建设。加快基层综合性文化服务中心建设，构建现代公共文化服务体系。开展文化强镇（区）创建。加快数字图书馆、数字文化馆等项目建设。加快“两城一园”文化创意产业园区建设。实施“版权兴业”工程，支持南方文化产权交易所中山服务中心、红木产权交易中心等项目建设。加大历史文化名城保护力度，加强古建筑、古村落、名人故居、优美乡村和非遗项目的保护开发。打造特色鲜明的文化旅游品牌，探索把“慈善万人行”打造成最具中山味道的新民俗节庆。扩大政府购买文化服务范围，鼓励社会力量举办各类文化活动。广泛开展全民健身活动。加快发展体育产业，鼓励社会力量举办各类体育赛事，办好国际马拉松邀请赛。发展校园足球。

　　维护社会平安稳定。加快完善立体化社会治安防控体系，加大力度创建“无医闹城市”、“场所无三害”、“无传销城市”、“无刑案村居”等系列“创无行动”，打造“平安中山”升级版，争创全国最平安城市。深化“全民禁毒”工程，完善社区戒毒康复模式，创建“全国禁毒工作示范市”。加强社会矛盾排查化解，重点解决涉农、涉劳资纠纷、涉环保、涉金融、涉房地产等问题。加强出租屋管理，提升服务流动人口水平。推进互联网安全监管能力建设。深入实施村居法律顾问制度。提升社区矫正安置帮教服务水平。做好安全生产和应急管理工作，加强隐患排查与综合治理，防范重、特大安全事故的发生。加大食品药品安全监管力度，推进全民创建食品安全城市和无假药城市工作，保障百姓饮食用药安全。

　　做好其它各项工作。做好扶贫工作，加大对欠发达村帮扶力度。加强残疾人服务设施建设。发展“智慧气象”，建设气象公园，提升气象现代化及防灾减灾能力。加强国防建设。做好人口计生、台务、民族宗教、妇女儿童、人防、地震、档案、方志等工作。

　　继续办好十件民生实事。今年全市统筹投入9.6亿元，集中力量办好十件民生实事。一是饮水安全工程，二是放心食品工程，三是健康惠民工程，四是交通畅通工程，五是城乡环境优化工程，六是关爱老人工程，七是关爱儿童工程，八是创业促就业工程，九是全民禁毒示范市创建工程，十是政务便民工程。

　　各位代表！做好今年各项工作，必须加强政府自身建设，更好发挥政府作用。一是建设服务型政府。坚持人民政府为人民，想民之所想，急民之所急。推进试点小区政务服务点建设，完善市行政服务中心微信平台，提升“智慧中山”APP应用功能。推进务工人员办证简易化。完善“12345”政务服务热线功能，加强政民互动沟通。二是建设法治政府。认真落实市人大及其常委会各项决议决定，主动加强同人民政协联系，认真办理人大代表建议和政协提案。坚持落实协商民主，广泛听取各民主党派、工商联、无党派人士和工青妇等人民团体意见。建立健全重大决策合法性审查制度，制定政府重大行政决策听证目录。统筹政府效能建设，探索试点开展重大活动行政成本评估。加强政务公开。推进行政复议委员会改革，建立健全行政复议开庭审理、公开听证制度。深入推进镇区综合行政执法改革。做好普法工作。三是建设高效政府。强化政府绩效管理，健全履职问责机制，坚决纠正慢作为、不作为、乱作为，确保政令畅通，令行禁止。四是建设学习型政府。大兴学习之风，形成学习进步的浓厚氛围。加大公务员队伍培训力度，拓展干部视野，推进思想解放，克服知识能力、队伍结构上的不足，打造一支高素质干部队伍。五是建设廉洁政府。严格落实中央八项规定，强化“三公”经费管理，加强对公务消费开支的监督检查，稳妥推进公务用车制度改革。加大审计监督力度，对公共资金、国有资产、国有资源和领导干部履行经济责任情况实行审计全覆盖。围绕项目招标、政府采购、土地出让等重点领域，开展监督检查，坚决查处各类违法违纪案件。

　　各位代表，新的蓝图已经绘就，新的征程已经开启。让我们在中共中山市委的坚强领导下，凝聚全体人民智慧，精诚团结、真抓实干、开拓进取，加快建设“三个适宜”更加美丽中山，再创新辉煌，再谱新篇章！
